# The influence mechanism of job satisfaction among Chinese rural teachers—a conservation of resources perspective

**DOI:** 10.3389/fpsyg.2025.1629885

**Published:** 2025-10-20

**Authors:** Hui Yu, Xiaona Gao, Zhi Yu

**Affiliations:** ^1^Huainan Normal College, Huainan, China; ^2^Jiaxing University, Jiaxing, China; ^3^Central China Normal University, Wuhan, China

**Keywords:** teacher job satisfaction, teacher professional development, resource conservation theory, multiple linear regression equation, rural teachers

## Abstract

Rural teachers are a cornerstone of China’s education system, entrusted with the critical mission of disseminating knowledge, fostering intellectual growth, and shaping the future in remote areas. However, their job satisfaction is influenced by a complex array of factors, which this study investigates through the theoretical lens of Conservation of Resources (COR) Theory. This study employed a cross-sectional design and analyzed data collected from 1,038 rural teachers in China. A multiple linear regression analysis was conducted to examine how variations in key resource domains impact their job satisfaction. The regression analysis revealed that perceived deficits in several resource areas significantly predicted lower job satisfaction. Specifically, we found that reductions in mental health resources, development and security resources, welfare-and-treatment resources, and interest-efficacy resources were all significantly associated with decreased satisfaction. Among these, a lack of interest-efficacy resources demonstrated the strongest negative association. The findings strongly support the application of COR Theory in this context, indicating that the loss of key personal and professional resources is a critical determinant of job satisfaction among rural teachers. We conclude that interventions aimed at enhancing job satisfaction should be structured around five core dimensions: fostering career interest and self-efficacy, ensuring professional development and security, promoting work–life harmony, improving the salary and welfare system, and safeguarding psychological health.

## 1 Introduction

As a professional cohort within the education sector, teachers’ job satisfaction critically drives their performance and profoundly impacts well-being, turnover rates, and long-term retention (Nguyen et al., 2020). Job satisfaction is essentially a psychological state that emerges when individuals compare the rewards they desire with those they actually receive, it is shaped by both external work conditions and internal motivations (Duraku et al., 2025). It influences teachers’ enthusiasm and autonomy, guides career planning, and affects classroom practice, ultimately shaping instructional quality, enriching pedagogical skills, and supporting students’ mental health (Eryilmaz et al., 2025).

Research on teachers’ job satisfaction in China spans nearly three decades and covers primary, secondary, and higher-education sectors. Rural teachers’ satisfaction not only reflects their professional motivation and instructional effectiveness, but also serves as a barometer for initiatives such as the New Era Basic Education Strong Teacher Plan, which aims to help rural teachers “to get started, stay, and teach well” (Ministry of Education of the People’s Republic of China, 2022; Huysman, 2018). Comparative studies in East Asia further highlight key predictors of satisfaction, using TALIS 2018 data, Niu et al., (2023) showed that teacher self-efficacy, emotional bonds with students, and team innovativeness are most influential. Liu et al. (2023) applied two-level hierarchical modeling to Shanghai data and found that both teacher-level factors (notably self-efficacy and intrinsic motivation) and school-level factors (especially campus safety) significantly affect satisfaction.

Building on these insights, the present nationwide survey maps the current landscape of rural teachers’ job satisfaction and identifies the core factors that shape it. The results will provide robust evidence to inform strategies for strengthening the rural teaching workforce and guiding more effective policy implementation.

## 2 Theoretical background

### 2.1 Rural teachers’ job satisfaction

The notion of “educational satisfaction” was first introduced by Taylor in 1912 and was later substantiated by Mayo and colleagues through the Hawthorne studies, which demonstrated that educators’ emotional and psychological states exert a profound influence on their teaching behaviors and overall school effectiveness (Wang et al., 2022). Building on these insights, Hoppock characterized educational satisfaction as teachers’ subjective perceptions of their work environment and the resulting sense of physiological and psychological fulfillment (Castro and Esposito, 2022). Although subsequent research traditions have nuanced this definition, there is broad consensus that educational satisfaction fundamentally reflects educators’ holistic affective responses to their professional context (Worrell et al., 2006). Interest in this construct endures because satisfied teachers are widely recognized as essential to the effective operation and long-term sustainability of educational institutions.

Research on the determinants of educational satisfaction has coalesced around two primary streams. The first examines internal factors, individual characteristics and intrinsic motivation, such as academic preparation, cultural literacy, expectations, and sense of fairness, alongside educational philosophy, professional identity, and commitment to teaching. Foundational theories in this domain include Maslow’s Hierarchy of Needs and Herzberg’s Two Factor Theory. The second stream emphasizes external factors, physical work environment, organizational climate, specific job content (e.g., curriculum design and pedagogical methods), and external incentives such as compensation structures and fair management practices (Alessandro et al., 2017). Within this framework, Hackman and Oldham’s Job Characteristics Model and Karasek’ s Job Demand–Control Model have become prominent tools for analyzing and enhancing teacher satisfaction.

Job satisfaction is the positive or negative appraisal individuals make of their work, directly shaping occupational engagement and overall life satisfaction (Weiss, 2003). In teaching, it refers to educators’ emotional responses to their professional roles (Skaalvik and Skaalvik, 2011) and is a critical determinant of effectiveness and commitment. Higher job satisfaction correlates with greater retention (Johnson, 2012), lower turnover (Skaalvik and Skaalvik, 2017), and serves as a key indicator of school performance (Tingting et al., 2022). A cross-national study of over 50,000 teachers in 34 countries found that improvements in satisfaction substantially bolster professional loyalty and intention to remain in teaching (Sims, 2017). Moreover, elevated satisfaction is linked not only to retention but also to improved student well-being, stronger school cohesion, and enhanced status of the teaching profession (Toropova et al., 2020). In rural contexts, teacher satisfaction is pivotal for staffing stability. Conversely, dissatisfaction can negatively affect instructional practices and undermine educational outcomes (Crisci et al., 2019). Further highlighting the role of contextual factors, recent studies in diverse settings have demonstrated significant relationships between job satisfaction and variables such as leadership styles (Juhji et al., 2025; Ibrahim et al., 2023) and professional development opportunities (Juhji et al., 2023), particularly during challenging periods like the pandemic. These findings reinforce the multifaceted nature of job satisfaction determinants across different educational environments.

### 2.2 Teacher job satisfaction and resource conservation theory

The Conservation of Resources Theory (CRT) was proposed by American psychologist Hobfoll in 1989 and is a fundamental meta-theory of human motivation, widely used in psychology and organizational behavior (Hobfoll, 2001). The core idea of this theory is that stress arises when valuable resources are threatened, depleted, or fail to deliver expected returns. When resources continue to drain or benefits are insufficient, individuals experience greater psychological stress and face a higher risk of burnout (Hobfoll et al., 2018). According to this theory, individuals are driven to acquire, protect, and build resources, such as material resources (e.g., money, time, property), psychological resources (e.g., self-esteem, social support, health, emotional energy, cognitive ability, self-concept), identity resources (e.g., organizational status), personality trait resources (e.g., skills, traits), and energy (e.g., time, knowledge). Hobfoll categorized these into four types of resources (Hobfoll, 1989), material resources (e.g., salary), environmental resources (e.g., work environment), personal trait resources (e.g., confidence), and energy resources (e.g., time, energy). Building on this framework, Ten Brummelhuis and Bakker proposed an improved classification that includes material condition resources, such as employment, family status, and social relationships, constructive resources, including skills, knowledge, resilience, and health, social support resources, such as emotional support, respect, guidance, and practical advice, and energy resources, such as emotions, cognitive ability, attention, and available time. This more detailed classification highlights the diversity of individual resources and reveals their dynamic interrelationships. In practice, these types of resources are interdependent. They continuously cycle and transform within the person-environment system, collectively maintaining physical and mental well-being as well as efficient performance. Scholars have extended the study of CRT by introducing two dynamic mechanisms, the “loss spiral” and the “gain spiral” (Hobfoll et al., 1990).

In a loss spiral, individuals with scarce resources become increasingly vulnerable to further depletion. As stressors outpace replenishment, rural teachers in particular experience diminished job satisfaction, heightened burnout, and waning professional enthusiasm. Conversely, a gain spiral occurs when resource-rich individuals leverage existing assets to acquire new ones, triggering an upward cascade of resource accumulation that reinforces well-being and performance. By centering on the fundamental drive to conserve resources and integrating these opposing cycles, CRT offers a causal framework for understanding employees’ psychological states and motivational drives. Within this model, strategies that support rural teachers’ resource maintenance, such as targeted professional development, peer mentoring, and workload management are critical levers for enhancing their job satisfaction and, ultimately, optimizing educational outcomes.

Drawing on domestic and international studies and CRT’s resource taxonomy, we identify four key categories that affect rural teachers’ job satisfaction (see Table 1).

**Table 1:** Classification of individual resources by Brummelhuis and Bakker.

Resource category	Resource component		Resource category
Material conditions resources	Marriage	Skills	Constructive resources
	Working	Knowledge	
	Family	Resilience	
	Social interaction	Health	
Social support resources	Emotional	Physical	Energy resources
	Respect	Cognitive ability	
	The guiding of important others	Attention	
	Advice and help	Time	

Material resources, such as salary and incentives (Yao and Zhen, 2024), which are fundamental to teachers’ decisions to “stay.” Development and security resources, including job resources and job security (Casely-Hayford et al., 2023), which provide the foundation for retention. Efficacy-emotion resources, such as self-efficacy (Richter et al., 2025), a supportive school climate (Admiraal and Rberg, 2023), and distributed leadership (Dicke et al., 2019). These directly shape teachers’ psychological states and emotional perceptions on the job. Psychological-energy resources, epitomized by work, life balance, family relationships, working hours, and overtime (Shahjahan et al., 2022; Wang and Lo, 2022; Xiaomei, 2023), which are especially vital for teachers in their marriage and childbearing years.

From an RTC perspective, these four factors align with CRT’s individual-resource categories. Material resources are rooted in the educational work environment and show relative stability.

Development and security resources, career opportunities and job protection, also originate in the work context but fluctuate over time, fitting the “social-support” category. Efficacy-emotion resources (self-efficacy, emotional well-being) constitute “personal-growth” resources, stable psychological traits essential to educators’ welfare. Work–life balance is a context-dependent subjective experience, classified as an “energy-regulation” resource.

Beyond these four pillars, individual characteristics, gender, fertility status, health, and family background, also modulate job satisfaction. Building on existing research, this study systematically examines how these diverse resources interact to shape rural teachers’ satisfaction mechanisms, offering new insights for human-resource management and professional-development strategies in education.

### 2.3 Research questions

The primary objective of this study is to assess rural teachers’ job satisfaction through the lens of Conservation of Resources Theory. Specifically, it identifies and analyzes the critical resources that underpin their professional well-being.

1. Welfare Resources. Compensation and benefits (e.g., salary, allowances, welfare packages).
2. Development and Security Resources. Supportive working conditions and opportunities for career advancement.
3. Work–Life Balance Resources. Mechanisms that facilitate harmony between teaching responsibilities and personal life.
4. Psychological Well-Being Resources. Professional identity, personal fulfillment, and quality of interpersonal relationships.
5. Interest-Efficacy Resources. Alignment of teaching tasks with teachers’ intrinsic interests and perceived competence.

The study examines how the acquisition and preservation of these resources enhance job satisfaction, and conversely, how resource depletion (e.g., inadequate remuneration, limited development prospects) undermines teachers’ motivation and satisfaction.

## 3 Methods

### 3.1 Sample

The data for this study came from a self-administered questionnaire, Rural Teachers’ Job Satisfaction, and the design of the questionnaire dimensions was based on the four categories classified by the Resource Conservation Theory. Five doctoral students majoring in pedagogy and an expert in pedagogy reviewed the reasonableness of the dimensional design and the accuracy of the questions’ content, and the questions with ambiguity in their presentation, and those that may be socially approving and inducing were revised and deleted before the questionnaire was finalized. The final version of the questionnaire was finalized. The survey questions covered personal information and job satisfaction. In order to improve the reliability and validity of the questionnaire, a small sample of 200 rural teachers in Anhui Province, China, was selected for the survey before the large-scale formal administration.

The coefficient of concordance (Cronbach’s alpha) of some items was greater than 0.8, and the overall reliability was good. Then a large-scale survey was conducted, and 1,300 rural teachers were randomly selected from rural areas of Anhui Province, China as subjects. After eliminating missing values and invalid samples, the effective sample size was 1,038. STATA18 was used to analyze the data by descriptive statistics, independent sample T test and multiple linear regression.

As shown in Table 2, the dependent variable “Rural Teachers’ Job Satisfaction” was expressed in the questionnaire as “How satisfied are you with your current job as a teacher.” The variable is scored on a 5-point Likert scale (“1″ = very dissatisfied, “5″ = very satisfied). The core independent variable “Individual resources” includes five items, welfare resources, development and security resources, life balance resources, mental health resources, and interest and efficacy resources, totaling 24 items. The consistency coefficient (Cronbach’s alpha) of all items was greater than 0.8, and the overall reliability was good, with a significant Bartlett’ s sphere test (*p* < 0.001, X2 = 13470.491), and the KMO = 0.947 > 0.7.5 common factors (with eigenvalues greater than 1) were extracted by exploratory factor analysis, and the cumulative variance explained rate was 68%, and the cumulative variance explained rate was 68% with a cumulative variance explained of 68.06%.

**Table 2:** Description of variables and descriptive statistics.

Variable type	Variable name	Description of variables	Average value	(statistics) standard deviation
Implicit variable	Job satisfaction	Satisfaction with current teachers’ work	3.158	1.087
Individual resource variables	Resources for welfare benefits	Fitted by 5 questions on salary, benefits, etc.	3.356	0.848
	Resources for development security	Fitted from 6 question items on career development opportunities, pre-school provision of training and learning resources	3.297	0.871
	Life balance resources	Fitted by 3 items such as reasonable work schedules, adequate school vacations and breaks, etc.	3.263	0.887
	Mental health resources	Fitted by 5 items on the importance of mental health status in schools, provision of counseling in schools, etc.	3.420	0.829
	Interest effectiveness resources	Fitted by 5 items such as interest in the current subject taught, professional competence is fully utilized in teaching, etc.	3.510	0.818
Individual characteristic variables	Distinguishing between the sexes	Male = 1, Female = 0	0.430	0.495
	Family residence	Urban = 1, rural = 0	0.437	0.496
	Years of experience	1–5 indicate less than 5 years, 5–10 years, 11–15 years, 16–20 years, and more than 20 years, respectively.	2.980	1.542
	Title	1–4 indicate no title, junior title, intermediate title, senior title respectively	2.633	0.901
	Reproductive status	Fertile = 1, infertile = 0	0.747	0.435

Individual characteristics include personal background information such as gender, family residence, years of work experience, job title, and reproductive status.

### 3.2 Measures

Since the dependent variable of the study is an ordered multicategorical variable, multiple linear regression was chosen to estimate the parameters of the influence of each factor on the job satisfaction of rural teachers using the least squares method, and the model of the regression equation was constructed as follows. Where satisfaction is the dependent variable indicating job satisfaction, X1i to X5i are the five core independent variables, X6i to X10i are the five individual characteristic variables, β1 to β10 are the corresponding regression coefficients, *α* is the constant term, and εi is the random error.


$$\begin{array}{l} \text{satisfactioni}=\alpha +\beta 1X1i+\beta 2X2i+\beta 3X3i\\ +\beta 4X4i+\beta 5X5i+\beta 6X6i+\beta 7X7i+\beta 8X8i\\ +\beta 9X9i+\beta 1\mathrm{0X}1\mathrm{0i}+\mathrm{\varepsilon i} \end{array}$$

## 4 Results

### 4.1 Distribution of individual resources of rural teachers

The distribution of rural teachers with different individual backgrounds on the five individual resources was analyzed by independent samples *t*-test to analyze the differences in the distribution of rural teachers with different individual backgrounds, and taking the mean scores of the five individual resources as the dependent variable, it was found that the satisfaction of male rural teachers was higher than that of female rural teachers on the dimension of mental health resources. In the dimensions of development security resources and welfare treatment resources, the satisfaction of rural teachers whose family residence is in rural areas is significantly higher than that of rural teachers whose family residence is in urban areas. In the dimensions of resources for interest and effectiveness and resources for welfare benefits, rural teachers who have not given birth are significantly more satisfied than those who have (see Table 3).

**Table 3:** Differences in the distribution of individual resources of rural teachers.

Test dimension	Clusters	Average value	(statistics) standard deviation	*T*-value
Mental health resources	Women	3.375	0.034	−2.007**
	Male	3.480	0.039	
Resources for development security	Countryside	3.352	0.036	2.298**
	Municipalities	3.227	0.041	
Resources for welfare benefits	Countryside	3.408	0.034	2.220**
	Municipalities	3.289	0.041	
Interest Effectiveness Resources	Infertile	3.591	0.049	1.902*
	Fertile	3.483	0.030	
Resources for welfare benefits	Infertile	3.445	0.051	1.999**
	Fertile	3.326	0.031	

***p < 0.01, **p < 0.05, *p < 0.1; this is a simplified table, presenting only significant differences.

### 4.2 Regression analysis of job satisfaction of rural teachers

In this study, the factors affecting rural teachers’ job satisfaction are divided into two levels. Individual resources and individual characteristics, and the factors affecting rural teachers’ job satisfaction are explored through multiple regression analysis. From the analysis of the multiple regression results of the factors affecting rural teachers’ job satisfaction in Table 4, it can be seen that there is a significant linear relationship between rural teachers’ job satisfaction and individual resources and individual traits (*F* = 116.00, *p* < 0.001), and the individual resource situation significantly predicts rural teachers’ job satisfaction. Individual resources and individual traits explain 53.04% of the rural teachers’ job satisfaction of the variance (*R*^2^ = 0.5304). The variance inflation factor was used to diagnose multicollinearity among the explanatory variables, and the variance inflation factor (VIF) of each variable was less than the threshold value of 10, which indicated that there was no multicollinearity problem among the variables.

**Table 4:** Results of regression analysis of factors affecting job satisfaction of rural teachers.

Variant	Model 1				Model 2			
	B	SE	Standardized beta	t	B	SE	Standardized beta	t
Resources for welfare benefits	0.405	0.038	0.315	10.750***	0.325	0.036	0.254	8.931***
Resources for development security	0.311	0.038	0.249	8.281***	0.282	0.036	0.226	7.858***
Life balance resources	0.217	0.034	0.177	6.436***	0.192	0.032	0.157	5.966***
Mental Health Resources	0.106	0.037	0.081	2.834**	0.109	0.036	0.083	3.059**
Interest Effectiveness Resources	0.067	0.034	0.051	1.992*	0.070	0.032	0.053	2.198*
distinguishing between the sexes					0.052	0.047	0.024	1.096
Family residence					−0.111	0.047	−0.051	−2.350*
Years of experience					−0.204	0.020	−0.289	−10.277***
Title					0.154	0.035	0.127	4.368***
Reproductive status					−0.135	0.060	−0.054	−2.264*
constant	−0.533	0.136		−3.925***	0.218	0.182		1.197
*R* ^2^	0.476				0.530			
Adjusted *R*^2^	0.473				0.526			

***p < 0.01, **p < 0.05, *p < 0.1; this is a simplified table, presenting only significant differences.

In the influence mechanism of rural teachers’ job satisfaction, all 5 categories of individual resources can significantly influence job satisfaction, and the increase of individual resources can significantly improve job satisfaction. According to the comparison of standardized regression coefficients, it is found that welfare treatment resources have the strongest influence among the 5 types of resources. Controlling other variables unchanged, for every 1-unit increase in welfare and treatment resources, development and security resources, life balance resources, mental health resources, and interest and efficacy resources, the job satisfaction of rural teachers increases by 0.405, 0.311, 0.217, 0.106, and 0.067 units correspondingly. Among individual characteristics, rural teachers’ family residence, years of working experience, job title, and maternity status have a significant effect on rural teachers’ job satisfaction. Among them, rural teachers living in the countryside have higher job satisfaction than those living in the city, for every unit increase in rural teachers’ working years, rural teachers’ job satisfaction decreases by 0.204 units correspondingly, for every unit increase in rural teachers’ job title, rural teachers’ job satisfaction increases by 0.154 units correspondingly, and the job satisfaction of non-childbearing rural teachers is higher than that of childbearing teachers. Gender has no significant effect on the job satisfaction of rural teachers.

According to previous studies, it is known that in the context of different individual characteristics, rural teachers have different resource reserves and rankings, and the influence of individual resources on their job satisfaction is also different. In order to test the heterogeneity of the influencing factors of rural teachers’ job satisfaction in the context of different individual characteristics, the interaction term between individual resources and individual characteristics is introduced in the regression model. According to Table 5, it can be seen that in Model 2, the interaction term of “mental health resources*family residence” (0.162**) and “interest and efficacy resources*family residence” (−0.128**) has a significant effect on the job satisfaction of rural teachers in terms of the influence of mental health resources and interest and efficacy resources. It can be seen that family residence plays a moderating effect on the effects of mental health resources and interest efficacy resources on job satisfaction, which can be seen in the fact that the effect of mental health resources on job satisfaction is greater when rural teachers live in urban areas than when rural teachers live in rural areas, and the effect of interest efficacy resources on job satisfaction is greater when rural teachers live in rural areas than when rural teachers live in urban areas. Situation. In Models 3 and 5, the interaction terms of “development and security resources*years of working experience” (0.048**) and “development and security resources*maternity status” (0.150*) have a significant effect on the job satisfaction of rural teachers, so it can be seen that working experience and maternity status play a moderating effect in the effect of development and security resources on job satisfaction, i.e., the interaction term of “development and security resources” and “development and security resources” has a significant effect on job satisfaction. It can be seen that years of working experience and fertility status play a moderating effect in the impact of development security resources on job satisfaction, i.e., the longer the working experience of rural teachers and the fact that they have already given birth to children, the greater the impact of development security resources on job satisfaction of rural teachers, and greater than the case of rural teachers with shorter working experience and not having children.

**Table 5:** Heterogeneity analysis of factors influencing rural teachers’ job satisfaction.

Variant	Model 1	Model 2	Model 3	Model 4	Model 5
	Adding interactions (gender)	Adding interactions (family residence)	Adding interactions (years of work experience)	Adding interactions (title)	Adding interactions (marital status)
Resources for welfare benefits	0.337*** (0.047)	0.309*** (0.049)	0.411*** (0.075)	0.290*** (0.111)	0.302*** (0.072)
Resources for development security	0.266*** (0.048)	0.292*** (0.047)	0.158** (0.074)	0.152 (0.114)	0.164** (0.070)
Life balance resources	0.266*** (0.048)	0.205*** (0.042)	0.250*** (0.065)	0.264*** (0.096)	0.113* (0.063)
Mental health resources	0.112** (0.046)	0.044 (0.047)	0.178** (0.072)	0.197* (0.111)	0.170** (0.071)
Interest effectiveness resources	0.091** (0.043)	0.123*** (0.042)	0.168** (0.067)	0.105 (0.103)	0.139** (0.065)
distinguishing between the sexes	0.025 (0.264)	0.060 (0.047)	0.052 (0.047)	0.051 (0.047)	0.053 (0.047)
Family residence	−0.112** (0.047)	−0.175 (0.259)	−0.108** (0.047)	−0.111** (0.047)	−0.109** (0.047)
years of experience	−0.203*** (0.020)	−0.203 *** (0.020)	−0.004 (0.088)	−0.203*** (0.020)	−0.209*** (0.020)
title	0.152*** (0.036)	0.155*** (0.035)	0.144*** (0.036)	0.191 (0.143)	0.161*** (0.035)
Reproductive status	−0.135** (0.060)	−0.136** (0.060)	−0.138** (0.059)	−0.135** (0.060)	−0.487 (0.310)
Interaction 1 (benefits package resources)	−0.027 (0.074)	0.047 (0.072)	−0.030 (0.024)	0.015 (0.041)	0.024 (0.083)
Interaction term 2 (resources for development guarantees)	0.038 (0.072)	−0.025 (0.073)	0.048** (0.023)	0.049 (0.040)	0.150* (0.081)
Interaction term 3 (life balance resources)	0.059 (0.065)	−0.035 (0.066)	−0.018 (0.021)	−0.027 (0.035)	0.104 (0.073)
Interaction term 4 (mental health resources)	−0.009 (0.073)	0.162 ** (0.072)	−0.024 (0.024)	−0.034 (0.041)	−0.075 (0.082)
Interaction term 5 (interest effectiveness resources)	−0.048 (0.065)	−0.128** (0.065)	−0.032 (0.022)	−0.013 (0.037)	−0.086 (0.075)
Constant	0.233 (0.214)	0.231 (0.218)	−0.431 (0.343)	0.110 (0.408)	0.486 (0.296)
*R* ^2^	0.531	0.534	0.536	0.532	0.534
Adjusted *R*^2^	0.524	0.527	0.529	0.525	0.528

***p < 0.01, **p < 0.05, *p < 0.1; this is a simplified table, presenting only significant differences. Interactions 1–5 are obtained by multiplying individual resources (resources for welfare benefits, resources for development and security, resources for life balance, resources for mental health, and resources for interest and efficacy) with individual characteristics (gender, family residence, years of work experience, job title, and fertility status), respectively.

## 5 Discussion

This study offers a nuanced analysis of the determinants of job satisfaction among rural teachers in Anhui Province, with implications for the broader Chinese context. First, consistent with Conservation of Resources Theory, all five resource dimensions, welfare benefits, developmental security, work–life balance, mental health, and interest-efficacy, emerged as significant positive predictors of overall job satisfaction. Notably, welfare benefits exhibited the largest standardized effect (*β* = 0.315), underscoring the critical role of material compensation in sustaining teacher morale in under-resourced settings. Although interest-efficacy resources yielded the smallest effect size (*β* = 0.051), they nonetheless made a unique contribution, highlighting that opportunities for meaningful pedagogical engagement and professional mastery foster intrinsic motivation even when extrinsic rewards are limited.

Second, these resource–satisfaction relationships echo national surveys showing that remuneration and career prospects are primary drivers of teacher retention in rural China (Li et al., 2024; Wang and Lo, 2022). By quantifying the relative weights of diverse resource categories, our findings provide policymakers with an evidence base for prioritizing subsidies and incentive structures. For example, increasing salaries and housing allowances may produce greater gains in satisfaction than equivalent investments in work–life initiatives, though the latter remain crucial for mitigating burnout and turnover among mid-career educators.

Third, subgroup analyses uncovered meaningful heterogeneity in resource effects. Mental health supports proved especially salient for teachers hailing from urban backgrounds, while interest-efficacy resources mattered most to those without childbearing responsibilities. These patterns suggest that non-local recruits, who often view rural posts as professional stepping stones, derive particular benefit from psychological services and avenues for skill enhancement. In contrast, native rural teachers may prioritize stable income and community integration over career-development perks. Tailoring resource allocation to these demographic profiles could optimize recruitment and retention strategies at both provincial and national levels.

Fourth, our results indicate that effective interventions should be informed by empirical resource assessments rather than by honor-system frameworks alone. Given regional disparities in economic development and educational infrastructure, context-sensitive measures are essential. Provinces experiencing acute teacher shortages might first bolster financial and housing incentives, whereas those with relatively stable staffing could focus on enhancing pedagogical autonomy and mental health supports.

In sum, this study demonstrates that comprehensive resource provisioning can simultaneously elevate rural teachers’ well-being and instructional quality. Future research should extend this framework through longitudinal tracking of resource changes and satisfaction trajectories, as well as comparative analyses across multiple provinces. Such work will further elucidate the causal pathways by which resource investments translate into sustained educational improvements and rural revitalization.

### 5.1 Implications for teacher education

Our study has implications for educational theory and policy. By studying teacher job satisfaction, we can replicate the operationalized approach to teacher job satisfaction (Torres, 2019).

#### 5.1.1 Activate interest-efficacy resources to ignite rural teachers’ intrinsic motivation

Educational interest serves as the primary engine driving rural teachers’ professional growth (Leichner et al., 2024), and interest-efficacy resources are vital for boosting their job satisfaction. To foster a self-reinforcing cycle of motivation and engagement, stakeholders should:

Minimize administrative barriers. Streamline regulations and reduce unnecessary paperwork so that teachers can devote more time and energy to activities aligned with their pedagogical passions.

Expand opportunities for pedagogical innovation. Provide clear, accessible pathways for rural teachers to participate in curriculum design, action research, and other interest-driven projects.

Reward excellence publicly. Establish regular recognition programs, such as teaching awards, performance grants, and peer-nominated commendations, to reinforce teachers’ sense of accomplishment and build their self-efficacy.

Build collaborative networks. Create and maintain communication platforms at multiple levels. Among rural schools, between schools and education authorities, and with external academic or professional organizations. These networks enable teachers to share best practices, consult with experts, and strengthen their professional identity and sense of belonging.

By deeply understanding and respecting rural teachers’ interests, and by actively nurturing their confidence in applying those interests, education systems can generate a virtuous cycle. Enriched interest-efficacy resources lead to higher job satisfaction, which in turn fuels further enthusiasm and innovation in rural education.

#### 5.1.2 Fortify development and security resources to cultivate a growth-oriented environment

Personal growth and career security are powerful motivators for rural teachers (Li et al., 2024), especially for non-local recruits and newcomers. To build a supportive ecosystem that enables teachers to flourish, rural schools and education authorities should. Modernize management practices. Align recruitment, evaluation, and promotion systems with the natural progression of teaching expertise. Streamline administrative processes to grant teachers greater autonomy over curriculum design and instructional methods.

Invest in professional development. Provide regular, high-quality training workshops and scholarship-supported academic exchange programs. Prioritize opportunities that address both subject-specific pedagogy and emerging educational technologies.
Establish structured mentoring. Pair novice teachers with experienced school leaders and veteran educators. These mentors should offer not only subject-matter guidance but also tacit insights into navigating the rural education context.
Revise performance evaluation. Center appraisal frameworks on demonstrated teaching excellence and student outcomes. Promote reasonable decentralization of decision-making, empowering teachers to innovate within their classrooms.
Diversify career pathways and protections. Create clear advancement tracks, such as lead-teacher roles, specialist positions, and school-administrative pathways, accompanied by robust job-security guarantees and competitive benefits.

By systematically strengthening these development and security resources, education stakeholders can deepen teachers’ sense of belonging and professional identity, thereby anchoring them as the backbone of sustainable, high-quality rural education.

#### 5.1.3 Prioritize work–life balance resources to sustain long-term engagement

Work–life balance is especially critical for rural teachers navigating marriage and parenthood. Given their heavier teaching loads, limited local resources, and elevated life-cost pressures, it is imperative that rural schools and education authorities support teachers’ personal well-being alongside their professional duties. To foster sustainable career development, they should:

Respect personal circumstances. Actively accommodate teachers’ family needs, such as childcare, eldercare, and personal emergencies, by offering flexible scheduling and access to essential life-support services.
Regulate workloads. Enforce reasonable limits on teaching hours and administrative tasks to prevent burnout. Optimize lesson-planning assignments and share teaching resources to lighten individual burdens.
Protect basic rights. Ensure that policies safeguard teachers’ statutory benefits and provide recourse against excessive overtime. A transparent system for grievance redress can help alleviate anxiety over unchecked work pressures.
Eliminate counterproductive competition. Discourage practices that pit teachers against one another through performance-only metrics. Instead, cultivate a collaborative culture that values collective success and mutual support.
Build a supportive environment. Introduce peer-support groups, wellness programs, and regular well-being check-ins, creating a climate in which teachers feel valued both as professionals and as individuals.

By embedding work–life balance at the heart of policy and practice, stakeholders can boost rural teachers’ job satisfaction, preserve their enthusiasm for teaching, and enhance the overall quality and stability of the rural education workforce.

#### 5.1.4 Strengthen welfare-benefit resources to underpin material security

Robust welfare packages are the bedrock of rural teachers’ career stability and satisfaction (Kidger et al., 2021), particularly for those balancing child-rearing responsibilities. To fully harness teachers’ commitment, policymakers and school leaders should:

Leverage dedicated funding streams. Channel public and private social funds into education-specific grants, such as a Rural Teacher Excellence Fund and an Education Innovation Fund, to finance pedagogical reforms, pilot programs, and individual career development.
Promote teacher-led innovation. Encourage rural educators to commercialize research findings and incubate education-related ventures, enabling them to secure additional income and gain professional recognition beyond their base salary.
Guarantee comprehensive social insurance. Ensure that schools or local governments provide full coverage of pensions, healthcare, and unemployment benefits in accordance with regional regulations, thereby safeguarding teachers’ basic living standards.
Expand targeted subsidies. Offer enhanced maternity, housing, and transportation allowances to reduce financial burdens and improve quality of life, especially for teachers in remote or understaffed areas.
Secure research funding access. Establish transparent policies for disbursing scientific-research grants, ensuring rural teachers have sufficient resources and infrastructure to conduct applied educational research.
Enhance compensation and working conditions. Regularly review and adjust salary scales to match cost-of-living increases, and invest in classroom infrastructure and teaching resources so that teachers can focus on instruction without undue material constraints.

By reinforcing welfare and incentive structures, education authorities can create an environment where rural teachers feel materially supported, enabling them to devote themselves wholeheartedly to teaching and the revitalization of rural education.

#### 5.1.5 Prioritize mental-health resources to protect teachers’ well-being

Rural teachers face mounting pressures, from increasingly complex teaching demands to fierce competition for positions, leading to significant mental-health challenges (Prabhu et al., 2024). Yet most existing services focus on students, leaving teachers underserved. Empirical findings further reveal that rural female teachers report lower satisfaction with available mental-health support than their male peers, underscoring an urgent need for tailored interventions. To address this gap, rural schools and education authorities should:

Build comprehensive support systems. Partner with local health departments, public hospitals, universities, and NGOs to establish dedicated psychological-service centers for teachers.
Deliver targeted counseling. Offer regular individual and group therapy sessions, led by qualified counselors, that address stress management, resilience building, and work-life integration.
Train school leaders and recruiters. Equip administrators with the skills to recognize early signs of distress, conduct empathetic conversations, and refer teachers to appropriate support.
Integrate wellness into professional development. Embed mental-health workshops, peer-support groups, and mindfulness training into existing training programs, ensuring teachers’ emotional needs are addressed alongside pedagogical growth.
Monitor and evaluate impact. Implement feedback mechanisms, such as anonymous surveys and focus groups to assess service effectiveness, with particular attention to gender-specific needs.

By strengthening and expanding mental-health services, especially for female educators, stakeholders can enhance teachers’ resilience, reignite their passion for teaching, and solidify the foundations for a thriving, sustainable rural education system.

### 5.2 Limitations and future research

Despite leveraging a large-scale survey of 1,038 rural teachers and applying rigorous regression analyses, several limitations of the current study should be acknowledged.

#### 5.2.1 Measurement consistency

Although we employed a standardized questionnaire grounded in Conservation of Resources Theory, individual interpretations of items may have introduced measurement variance (Richter et al., 2022). Future work should undertake formal psychometric validation, such as confirmatory factor analysis and test, retest reliability, to ensure that each of the five resource dimensions (welfare benefits, development security, life balance, mental health, interest-efficacy) is assessed with maximal precision and comparability.

#### 5.2.2 Cross-sectional, provincial scope

Our data were collected at a single time point and primarily reflect conditions in Anhui Province. Consequently, causal inferences regarding how resource fluctuations drive changes in satisfaction are limited. Additionally, regional policy environments and cultural norms differ substantially across China, so findings may not generalize to all rural settings. Longitudinal, multi-province studies are needed to trace how shifts in resource provision (e.g., new incentive schemes) affect teacher satisfaction over time.

#### 5.2.3 Sample representativeness

While our 1,038 respondents provide robust statistical power, the sampling frame may under-represent teachers in remote or ethnic-minority areas. Groups whose resource needs and satisfaction profiles often diverge from the provincial average. Future research should employ stratified sampling to ensure inclusion of these under-studied subpopulations, thereby capturing the full spectrum of rural teacher experiences.

#### 5.2.4 Unmeasured influences

Our model explained over 50 percent of the variance in job satisfaction, yet key factors, such as personal values, professional identity, and family support, were not directly measured. Qualitative or mixed-methods designs (e.g., focus groups, in-depth interviews) could uncover these latent drivers and illuminate how they interact with material and psychological resources to shape satisfaction.

##### 5.2.4.1 Recommendations for future research

Standardize and validate the five-resource scale at the national level, enabling policymakers to benchmark satisfaction and tailor interventions appropriately.

Expand surveys longitudinally across multiple provinces to evaluate the impact of evolving support programs (e.g., housing subsidies, honor systems) on teacher retention and well-being.

Adopt mixed-methods approaches in under-represented regions to surface context-specific needs particularly for female teachers and non-local recruits whose satisfaction hinges more on mental-health and interest-efficacy resources (McMahon-Morin et al., 2025).

Integrate unmeasured factors by incorporating items on professional identity and family support into future questionnaires, thus providing a more holistic map of rural teacher satisfaction drivers.

By addressing these gaps, subsequent research can offer more precise, generalizable guidance for enhancing job satisfaction, and thereby performance, retention, and well-being, among China’s rural teachers.

## Data Availability

The raw data supporting the conclusions of this article will be made available by the authors, without undue reservation.
